# The Classic Three-Month Post-Operative Adaptation Phase in Foot and Ankle Surgery—An Expert Perspective

**DOI:** 10.3390/jcm12196217

**Published:** 2023-09-26

**Authors:** Simone Santini, Andrea Marinozzi, Mario Herrera-Pérez, Sergio Tejero, Martin Wiewiorski, Cesar de Cesar Netto, Alexandre Leme Godoy-Santos, Victor Valderrabano

**Affiliations:** 1 Swiss Ortho Center, Swiss Medical Network, Schmerzklinik Basel, Hirschgässlein 15, 4010 Basel, Switzerland; s.santini@unicampus.it; 2 Department of Orthopaedic and Trauma Surgery, University Campus Bio-Medico of Rome, Via Alvaro del Portillo 200, 00128 Rome, Italy; 3 Foot and Ankle Unit, Orthopaedic Department, Universidad de La Laguna, 38200 San Cristóbal de La Laguna, Spain; 4 Foot and Ankle Unit, Orthopedic Surgery and Traumatology Service, Hospital Universitario Virgen del Rocío, Av. Manuel Siurot s/n, 41013 Sevilla, Spain; 5 WinOrtho, Technikumstrasse 61, 8400 Winterthur, Switzerland; 6 Division of Orthopedic Foot and Ankle Surgery, Department of Orthopedics, Duke University, Durham, NC 27708, USA; 7 Hospital Israelita Albert Einstein, Av. Albert Einstein 627, Sao Paulo 05652-900, Brazil

**Keywords:** ankle, foot, foot and ankle surgery, post-operative, adaptation phase, orthopaedics

## Abstract

Foot and ankle disorders are a common reason for orthopedic surgical intervention. After surgery, specific precautions such as partial weight bearing or complete unloading, and the use of walking aids, coupled with a period of rest, are usually implemented to ensure the surgical outcome. However, when these aids are discontinued and the patients resume load increase and normal daily activities, they may enter a transitional phase characterized by inflammation, swelling, and pain. We call this phenomenon the “classic three-month post-operative adaptation phase” (POAP). It is essential to differentiate this physiological transition phase from other conditions, such as from the immediate post-surgical inflammation, complex pain regional syndrome, or an infection. The objective of this expert opinion is to describe and raise medical awareness of this evidence-based phenomenon, which we commonly observe in our daily practice.

## 1. Introduction

Foot and ankle (F&A) disorders are among the most frequent reasons for orthopedic examination [1,2]. Since they are predominantly deformities, a large number of diagnoses result in surgery. The acute inflammatory response is crucial for the healing process of tissues [3]. Inflammatory markers play a crucial role in communicating with dormant stem cells, prompting them to become active and contribute to the tissue’s regenerative process [3]. When skeletal muscles are injured, by injury or surgery, inflammation initiates within approximately 6 h after the injury, causing an upsurge in the expression of inflammatory cytokines and chemokines like tumor necrosis factor alpha (TNF-α), macrophage inflammatory protein-1 (MIP-1), and monocyte chemoattractant protein-1 (MCP-1) [4]. Neutrophil and macrophage populations reach their peak levels at 24 h and 72 h after the injury, respectively, and the inflammatory response typically subsides within 7 to 10 days. Similarly, bone injuries lead to a swift response involving cytokines such as TNF-α, and interleukin-1, -6, and -11 (IL-), which attract neutrophils and macrophages to the site of injury. This inflammatory process is usually resolved within seven days after the bone injury occurs [5,6]. In the postoperative period, a series of precautions, including weight-bearing modifications and using walking aids associated with a period of substantial rest, ensure that the tissues have time to heal and the patient is essentially pain-free [6]. When these aids are removed, 6 weeks is the average for elective cases, up to 12 for severe trauma, and then the patient returns to normal daily activities. A transitional phase of physiological inflammation, swelling/edema, redness, and pain may be observed for a further 1–3 months. We define this phenomenon as the “classic three-month post-operative adaptation phase” (POAP), i.e., adaptation as the foot adapts from partial/none to full weight-bearing. This condition can be misdiagnosed several times by surgeons, general practitioners, physiotherapists, and patients with complex pain regional syndrome (CPRS) or those with an infection.

CRPS poses a significant challenge for both doctors and patients due to its complex nature and the tendency for chronicity and relapses, which can lead to substantial disability [7]. It is a complex condition to diagnose and treat, necessitating regular monitoring to ensure progress is being made [8]. CRPS following an F&A surgery significantly affects the ability to walk, leading to a considerable loss of productivity as patients are unable to commute to their workplace [9]. The prevalence of CRPS after foot and ankle injuries and surgical procedures is estimated to be approximately 13% [10].

Timely diagnosis and treatment are crucial in order to prevent long-term or permanent disability. The clinical characteristics of CRPS, such as spontaneous pain, swelling, heightened sensitivity to pain, changes in temperature or sweating, abnormal motor function and autonomic changes, serve as defining features of the disease [11]. The management of CRPS is a subject of controversy and encompasses various approaches such as medication, physical therapy, regional anesthesia, and neuromodulation [12].

F&A orthopedic surgery is a specialized field that involves treating a wide range of conditions, including painful deformities, trauma lesions as fractures or soft-tissue damages, soft-tissue surgeries, issues related to implants, diabetic foot disorders, infections, tumors, and others [13]. Each surgical day presents a diverse array of operative wounds, varying in terms of contamination level, thereby subjecting the surgeon to different infectious risks for each individual case [14]. The incidence of postoperative infections following F&A surgery has been reported to be as high as 6.5% [15]. Anatomically, the lower extremity, particularly the F&A, may have a heightened susceptibility to developing such infections [13].

Therefore, it is of paramount importance to be able to distinguish these two major complications of F&A surgery, CRPS and infections, as well as the first-weeks post-surgical inflammation, from the “three-month POAP”. Our purpose is to describe and increase medical awareness of this evidence-based phenomenon that we see on a daily basis and that we define as the “classic three-month POAP”.

## 2. Most Frequent Surgeries in F&A

The most common F&A procedures depend on various factors, including geographical location, population demographics, and prevalent foot conditions. According to the Swedish national quality register for F&A surgery (Swefoot) [16], the percentage distribution of each surgery type in the forefoot group during the year 2020 was as follows:

Hallux valgus with 1793 surgeries (42%); hammertoes with 1374 surgeries (32%); hallux rigidus with 637 surgeries (15%); Morton’s neuroma with 240 surgeries (6%); Taylor’s bunion with 194 surgeries (5%) [16].

Regarding the percentage distribution in the hindfoot group, it was as follows: osteoarthritis (OA) in the hindfoot/midfoot 342 (31%), flatfoot deformity 134 (12%), ankle instability 170 (15%), peroneus tendon pathology 133 (12%), insertional Achilles tendinopathy 124 (11%), cavovarus deformity 73 (7%), lateral calcaneal ridge hypertrophy 46 (4%), foot drop 51 (5%), Achilles tendon rupture (>4 weeks) 28 (3%), and Achilles tendinopathy 15 (1%) [16].

In our experience, the three-month adaptation phase is independent of the location of the surgery, appearing to be most severe in surgery that requires longer immobilization time or partial weight-bearing.

## 3. Physiopathology and Pathomechanics

After a high amount of F&A orthopedic surgery, mobility and foot contact with the ground are generally restricted partially or totally in the first postoperative 6 weeks, sometimes even 8 weeks. Crutches, walkers, casts, scooters, postoperative shoes, and other methods are sometimes used to let the tissues heal and to protect the surgical outcome [6]. The patient is advised to rest and keep elevating the limb in this first 6-week postoperative phase. Nonsteroidal anti-inflammatory drugs (NSAIDs) and physiotherapy are prescribed. Cautious physiotherapy consists of lymphatic drainage, light passive mobilizations or/and, when indicated, isometric strengthening of major muscle groups of the foot and ankle. Some swelling and hypersensitivity of the operated area are invariably present. Pain is more intense the days after the surgery, but is generally well controlled by the use of NSAIDs after a few days.

At 6–8 weeks on average, the patients come for their first postoperative check-up. After the confirmation of tissue healing, e.g., bone healing of a fracture, an osteotomy, a total ankle arthroplasty (TAA), or arthrodesis by X-rays, the patient is from then on allowed to remove the deambulatory aids gradually and to use normal shoes, and is encouraged to restart their daily activities progressively. Physiotherapy usually becomes more aggressive: full-weight bearing, gait training, eccentric strengthening, proprioception. The foot starts to work again.

The bone (fracture, osteotomy, TAA, arthrodesis) is healed, however still too weak to support the full body load. Furthermore, soft tissues, ligaments, capsules, and tendons are all retracted from the inactivity period. The load increase due to the weight-bearing causes microlesions in the tissues and therefore a physiologic second peak of healing inflammation, with consequent swelling, redness, and pain. Imaging exams show no delayed union or any pathology.

It has already been described that 3 months after a total ankle arthroplasty (TAA) surgery, patients experiment with a transient negative rehabilitation phase, scoring worse in both ranges of motion (ROM) and power exercises compared to pre-operative levels. With time, we can assist in an inversion of this trend, with improvements in rehabilitation results after 6 months [17].

There are several possible explanations for this negative phase of rehabilitation, during which patients have not yet reached their normal level of functioning. These reasons could include (1) the classic three-month POAP described in this paper, (2) the decrease in patients’ functionality and muscle inactivity caused by the necessary immobilization of the ankle using a walking boot and crutches for the initial 6-week period after surgery, or (3) the unidentified factors that may also contribute to this outcome [17].

**Figure 1:**
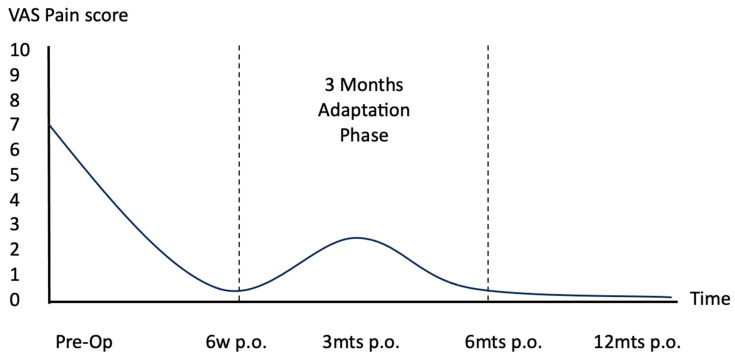
**The post-operative rehabilitation curve in foot and ankle orthopedic surgery**. Pre-Op: preoperative; 6w p.o.: 6 weeks postoperative; 3mts p.o.: 3 months postoperative; 6mts p.o.: 6 months postoperative; 12mts p.o.: 12 months postoperative. Dotted area: The Classic Three-Month Post-Operative Adaptation Phase.

The post-operative convalescence curve (Figure 1) visually describes this phenomenon. Pre-operatively, the patient is experiencing pain and discomfort at the maximal level. At the six-week follow-up, due to the immobilization/protection/NSAIDs, the patient is at the lowest point of the curve, reporting no pain. Variable degrees of swelling might be appreciated. At the three-month follow-up, the patients present to us with the typical set of signs and symptoms of the adaptation phase. In our experience, this is a self-limiting condition, the natural evolution of which is to resolve spontaneously within 6–12 months following surgery, when the patients will report well-being again.

## 4. The Clinics of the Classic Three-Month Post-Operative Adaptation Phase

At the three-month follow-up, the patient experiences the lowest level of satisfaction from the surgery. The three key signs of this adaptation phase are pain, swelling, and redness.

The pain typically decreases at rest and is more intense during walking or physiotherapy, and is is invariably present.

**Figure 2:**
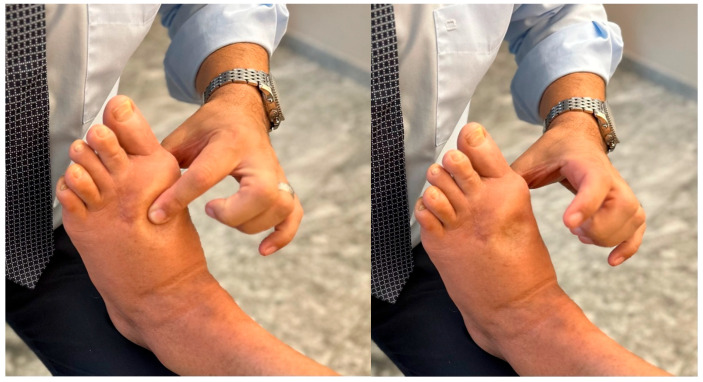
**Classic swelling/lymphatic edema in the three-month post-operative phase**. Pitting edema in a patient at the three-month post-operative follow-up after a Lapidus procedure and metatarsal osteotomies.

The swelling can range from mild to pitting (Figure 2), and can be so massive in some cases that it causes limitation to the F&A range of motion, with consequent pain following mobilization attempts. Redness can be locally limited to the surgical incision’s surrounding area or diffuse due to the lymphatic pressure to the skin. All these signs may easily lead to misdiagnosis with an infection or a CPRS. Surgical site infections (SSI) manifest either within 30 days following surgery or within 90 days if there is an implantation of prosthetic material. The complete absence of elevation in inflammatory lab values (VES and PCR) and sinus presence, along with any fever and systemic symptoms may be of help in the differential diagnosis between infection and this 3-month postoperative adaptation inflammation. CPRS, likewise, manifests itself with a broader autonomic symptom spectrum, and Budapest criteria can help the surgeon with their differential diagnosis [18].

The severity of 3-month POAP depends on several factors. In our experience, female sex, smoking, advanced age, preexisting osteopenia/osteoporosis, high body max index (BMI), and unaddressed deformities on the same side are all worsening factors.

## 5. Conservative Treatments

In order to accelerate the healing process and alleviate the swelling and pain in the 3-month POAP, we regularly implement the following conservative measures:

1. Adjusting load and activity level: Modifying the amount of weight-bearing or activity level to a subjective accepted level is important. This may involve the temporary use of crutches, walkers, or other assistive devices to offload weight from the foot, allowing the injured area to heal without excessive stress.

2. Wearing more protective shoes/insoles: Utilizing footwear that provides adequate support, cushioning, and protection can help to reduce pain. Customized insoles or orthotics may be recommended to provide additional support and improve foot mechanics [19].

3. Elevation of the foot/leg: Raising the foot or leg above the heart level helps to reduce swelling by promoting better circulation and reducing fluid buildup [20].

4. Manual lymphatic drainage: Lymphatic drainage techniques involve gentle massages or movements that stimulate the lymphatic system, promoting the removal of excess fluid from the affected area. This can help to reduce swelling and enhance the healing process [21].

5. Compression stockings: Applying compression stockings or wraps helps to compress the tissues, improve blood flow, and reduce swelling. This can aid in promoting healing and relieving discomfort [22].

6. Healing improvement medication: Vit. D3, Ca^2+^, Vit. C, protein to reach nutrition goals, and others.

Adequate vitamin D levels may enhance the rate of bone healing, strengthen the bone, and reduce the risk of delayed union or nonunion [23]. Vitamin D also plays a role in muscle function and strength. After F&A ankle surgery, maintaining muscle strength and function is essential for proper gait and mobility during the recovery process [24]. Vitamin D deficiency has been associated with muscle weakness and an increased risk of chronic pain and depressive symptoms. Adequate vitamin D levels may contribute to improved pain management and overall mood during the recovery period after surgery, and may help to reduce the likelihood of postoperative pain and improve the patient’s emotional well-being [25]. Vitamin D is involved in modulating immune function, and adequate levels may support the body’s defense mechanisms during the recovery phase after surgery. This may help to reduce the risk of postoperative infections and support overall healing [26]. Antioxidant vitamin intake (e.g., vitamin C, vitamin E, retinol, and β-carotene) could improve physical performance in the elderly. There are significant positive correlations between most antioxidants, particularly vitamin C, and higher skeletal muscular strength [27].

Protein stands as a crucial macronutrient; it plays a necessary part in every aspect of the healing process, encompassing the growth of fibroblasts, the creation of collagen, the development of new blood vessels (angiogenesis), and immune function support [28,29].

7. Cryotherapy: Applying a cold substance, like a cold curd wrap or ice, to the skin around swollen soft tissues and joints is believed to work by decreasing the temperature within the affected area. This decrease in temperature helps to alleviate pain by reducing the speed at which nerves transmit signals, and by immediately causing the blood vessels to constrict. This constriction reduces vascular spasms and slows down the flow of blood, resulting in a decrease in tissue swelling [30].

8. Sea salt bath: Soaking the foot or leg in a warm sea salt bath can have a soothing effect and promote healing [31].

By incorporating these conservative measures, we aim to facilitate the healing process, minimize pain, and enhance overall recovery.

## 6. Conclusions

The three-month POAP is a physiological transition phase in which the foot passes through a second peak of healing inflammation, adapting to the normal load of the foot and ankle. It is of paramount importance to know its characteristics and be able to differentiate it from other pathological conditions.

All the symptoms are self-limiting; nonetheless, several conservative treatments may be used to reduce the recovery time.

## Data Availability

This is an expert opinion. It is not a study.
